# Integrin-Functionalised Giant Unilamellar Vesicles via Gel-Assisted Formation: Good Practices and Pitfalls

**DOI:** 10.3390/ijms22126335

**Published:** 2021-06-13

**Authors:** Mariem Souissi, Julien Pernier, Olivier Rossier, Gregory Giannone, Christophe Le Clainche, Emmanuèle Helfer, Kheya Sengupta

**Affiliations:** 1 Aix Marseille Univ, CNRS, Centre Interdisciplinaire de Nanoscience de Marseille (CINAM), Turing Centre for Living Systems, 13009 Marseille, France; souissi@cinam.univ-mrs.fr; 2 Université Paris-Saclay, CEA, CNRS, Institute for Integrative Biology of the Cell (I2BC), 91198 Gif-sur-Yvette, France; Julien.PERNIER@i2bc.paris-saclay.fr (J.P.); Christophe.LECLAINCHE@i2bc.paris-saclay.fr (C.L.C.); 3 Univ. Bordeaux, CNRS, Interdisciplinary Institute for Neuroscience, IINS, UMR 5297, 33000 Bordeaux, France; olivier.rossier@u-bordeaux.fr (O.R.); gregory.giannone@u-bordeaux.fr (G.G.)

**Keywords:** in vitro reconstitution, liposome, functionalised GUVs, adhesion

## Abstract

Giant unilamellar vesicles (GUV) are powerful tools to explore physics and biochemistry of the cell membrane in controlled conditions. For example, GUVs were extensively used to probe cell adhesion, but often using non-physiological linkers, due to the difficulty of incorporating transmembrane adhesion proteins into model membranes. Here we describe a new protocol for making GUVs incorporating the transmembrane protein integrin using gel-assisted swelling. We report an optimised protocol, enumerating the pitfalls encountered and precautions to be taken to maintain the robustness of the protocol. We characterise intermediate steps of small proteoliposome formation and the final formed GUVs. We show that the integrin molecules are successfully incorporated and are functional.

## 1. Introduction

Adhesion of cells to their micro-environment is essential to most life forms. Cells adhere to each other and to the extracellular matrix (ECM), and they adapt their architecture and function to the micro-environment they access through adhesion [1]. Cell-matrix adhesion, mediated by cell adhesion molecules (CAMs), plays a critical role in this process of adaption. CAMs often cluster together and form special structures that mediate adhesion and mechanosensing/mechanotransduction. An important example is that of integrin-dependent adhesions, including early Nascent Adhesions and mature Focal Adhesions (FAs), which are major mechanosensitive complexes made of integrin transmembrane receptors that mechanically couple the ECM to the acto-myosin cytoskeleton, via actin binding proteins [2,3]. The mechanosensitivity of integrin adhesive structures allows cells to adapt their adhesion to the internal force of the acto-myosin cytoskeleton and to the physical properties of the ECM [1,3,4,5,6].

Integrin molecules establish the crucial molecular links in FAs that connect the extra- and intracellular environment. They are ubiquitous transmembrane proteins that transmit acto-myosin forces via their intracellular domain connected to actin-binding proteins, to their extracellular domain linked to ligands presented within the cell microenvironment. Interestingly, the chemical affinity of individual integrins [5,7,8] and the stability of the bond aggregates can be strongly influenced by acto-myosin forces [4,9]. Crucially, individual interactions of integrin extra- and intracellular domains with their binding partners are so dynamic that force transmission should be possible only through cooperation. FAs represent just such cooperative structures.

Early theoretical work showed that the cell membrane could play a crucial role in orchestrating such cooperative effects [10,11], and recently there has been renewed interest in physics of membranes, in particular, membrane fluctuations [12,13,14,15,16,17,18,19,20], domain formation [21,22,23,24,25,26,27,28,29,30], interaction with actin [31,32,33,34,35], role of surface polymers [36,37,38,39], and topography [40,41,42,43], often in the context of adhesion. Recently, glycocalyx-induced topography was recognized to be a relevant physical factor in cells [39], vindicating earlier model membrane studies [36,44]. Considerable progress in understanding physical principles of membrane adhesion has been achieved using cell models such as giant unilamellar vesicles (GUVs) (see [45,46,47,48] for a review), detailing the process of nucleation (seeding of new domains) and growth in a variety of scenarios [49]. However, experiments using physiological transmembrane molecules have been relatively rare due to the difficulty in incorporating correctly folded amphiphilic transmembrane proteins into a reconstituted membrane.

Giant unilamellar vesicles (GUVs) have proved to be indispensable for understanding the biochemistry and biophysics of membrane-bound proteins [42,50]. Very early experiments depended on spontaneous formation of GUVs in lipid–water mixtures, but this technique is not very robust, and yields are poor. The introduction of electro-formation [50,51] standardised the process of “swelling” vesicles. The major advantage of both these approaches is that there is no possibility of contamination from any other material. Various kinds of fluidic-based techniques have been developed more recently [52,53,54] and provide high yield and very good control over the GUV architecture. However, the presence of an oil phase during formation may introduce unwanted material into the hydrophobic chain region of the GUV bilayers. Gel-assisted formation is a more recently introduced technique, where the lipids are hydrated over a polymer layer [48,55,56,57,58]. The advantage of this technique is its simplicity, as it requires practically no instrumentation in contrast to the fluidics techniques. Further advantages of gel-assisted swelling are that, unlike electro-swelling, vesicle formation is fast, decreasing the risk of lipid and protein degradation, there is no restriction in lipid composition, and a buffer containing physiological amounts of salt can be used [48]. The polymer of choice for the gel layer is polyvinyl alcohol (PVA), which was shown not to be incorporated into the GUVs, unlike some other polymers [48].

Incorporating a transmembrane protein to a GUV is an additional challenge [59]. Streicher et al. pioneered the incorporation of integrins into GUVs [60] and demonstrated their adhesive functionality. More recent works have reconstituted functional integrins into small unilamellar proteoliposomes [61] as well as GUVs [62], the latter focusing on ligand binding from solution, rather than adhesion to a surface.

Early works on membranes reconstituted with purified integrins, using GUVs and supported lipid bilayers (SLBs) system, have shown that ligand-bound integrins spontaneously form domains [50,63] which grow, and become more bond-dense under force transmitted through the membrane, purely due to the elasto-thermodynamics of the system [50]. However, in these experiments, the integrins were on SLBs and their ligands on the GUVs. Similar adhesive behaviour was demonstrated in the inverse system where the integrin is incorporated into the GUVs and the ligands are immobilised on a surface [60].

Both these systems provided insight into integrin-mediated adhesion, but crucial questions, such as the impact of changing ligand and/or receptor concentration on cooperative adhesion kinetics or the role of partner actin binding proteins such as talin, remain to be explored. This is partly because producing GUVs containing correctly functional transmembrane integrins remains a challenge. The aim of this work is to identify the critical parameters that, so far, have limited the production of integrin-containing GUVs. We have systematically explored the fabrication of functional liposomes carrying the integrin αIIbβ3 extracted from blood platelets using gel-assisted swelling, and we explored the parameters that control the fabrication process. We compare gel-assisted swelling with electro-swelling, and we demonstrate adhesive function of the integrin-containing GUVs.

## 2. Materials and Methods

### 2.1. Chemicals

The lipids: SOPC (1-stearoyl-2-oleoyl-sn-glycero-3-phosphocholine), DOPC (1,2- didecanoyl-sn-glycero-3-phosphocholine), DOPE-PEG(2000) Amine (1,2-dioleoyl-sn-glycero- 3-phosphoethanolamine-*N*-[amino(polyethylene glycol)-2000] (ammonium salt)), Egg PE (L-α-phosphatidylethanolamine (Egg, Chicken)), Egg PC (L-α-phosphatidylcholine (Egg, Chicken)), 18:1 Dansyl PE (1,2-dioleoyl-sn-glycero-3-phosphoethanolamine-*N*-(5-dimethylamino-1-naphthalenesulfonyl) (ammonium salt)), and DOPE-RGD (1,2-dioleoyl-sn-glycero-3-phosphoethanolamine-*N*- [4-(p-(cysarginylglycylaspartate-maleimidomethyl) cyclohexane-carboxamide] (sodium salt)) were purchased from Avanti Polar Lipids (Alabaster, AL, USA) either as powder or in chloroform, subsequently dissolved/diluted in chloroform to a concentration of 1 mg/mL, and stored at −20 ${}^{\circ }$C.

Triton X-100 detergent was purchased from Roche Diagnostics (Mannheim, Germany), Bio-beads (SM2, 25–20 mesh) from Bio-Rad (Hercule, CA, USA), Tri-peptide Arg-Gly-Asp (RGD) from Sigma-Aldrich/Merck (Darmstadt, Germany), and Polyvinyl alcohol (PVA, MW 145,000) from Merck (Darmstadt, Germany). Phosphate buffer saline (PBS) in powder form from Sigma-Aldrich/Merck (Darmstadt, Germany) was dissolved in deionized water to a concentration of 1× (150 mM, pH 7.4). Bovine Serum Albumin (BSA, Darmstadt, Germany) was resuspended in PBS buffer to a concentration of 10 mg/mL. Osmolarites of the solutions were measured with an osmometer (Gonotec OSMOMAT 030) and adjusted to values between 200 and 300 mOsm when needed in order to maintain correct osmotic balance.

### 2.2. Integrin Purification and Labeling

Integrin αIIbβ3 was purified using a previously published protocol [64]. Briefly, integrin was extracted from outdated human platelets (from French Blood Etablishment (EFS)) with 1% Triton X-100 and purified via affinity chromatography over Concanavalin A, Heparin Sepharose, and KYGRGDS-columns (HiPrep 16/60 Sephacryl S-300 HR, Sigma-Aldrich). The functionality of the integrins was verified by checking the ability to fix on the KYGRGDS column upon addition and removal of 2 mM MnCl${}_{2}$. Proteins were isolated in pure inactivated form by size exclusion chromatography Sephacryl S300 1660 (GE Healthcare). Purified integrins were labelled with Alexa Fluor 546 succinimidyl ester following the manufacturer’s protocol (Thermofisher). Integrin concentration was measured by reading the absorbance at 280 nm using a calculated extinction coefficient of 238,515 M${}^{-1}$ cm${}^{-1}$. The concentration of the Alexa Fluor 546 dye was similarly measured from the absorbance at 554 nm using a calculated extinction coefficient of 112,000 M${}^{-1}$ cm${}^{-1}$. The labeling rate was derived from the ratio of the dye and integrin concentrations. The batch of labeled integrin used here is at a concentration of 11.2 mg/mL, with a 58% labeling rate. Integrins were stored at −80 ${}^{\circ }$C in a buffer containing detergent (20 mM Hepes pH 7.5, 150 mM NaCl, 1 mM CaCl${}_{2}$, 1 mM MgCl${}_{2}$, 0.1% Triton X-100, and 0.01% NaN${}_{3}$).

### 2.3. Functionalised Surfaces

Square glass coverslips (24 × 24 mm${}^{2}$, 0.17 mm thick, Hecht assistant, Germany) were used as substrates with various coatings. Before use, the coverslips and the observation chambers were cleaned using a surfactant-based procedure (30 min with 2% Hellmanex detergent in an ultrasonic bath (Digital Ultrasonic Cleaner), 10-times rinsing with milliQ (MQ) water and 30 min washing with MQ water in the ultrasonic bath, followed by another round of surfactant and water washing, and a final 30 min washing with MQ water in the ultrasonic bath followed by 10 times rinsing with MQ water; storage in MQ water until use). After preparation, all types of substrates were mounted in a made-to-order observation chamber.

Passivated surfaces were prepared by adsorbing BSA to cleaned glass surfaces: 2% BSA (*v*/*v*) in PBS was spread onto the coverslip and incubated for 30 min at RT, before rinsing 10 times with PBS.

SLBs were prepared according to the protocol in Ref. [65]: a Langmuir film-balance (Nima, Coventry, UK) was used to apply Langmuir–Blodgett/Langmuir–Schaefer (LB/LS) techniques. Both layers are composed of SOPC + 2% DOPE-PEG + 1% Dansyl-PE + 5% DOPE-RGD). After preparation, SLBs were passivated with BSA.

### 2.4. Preparation of Small Unilamellar Vesicles Containing Integrins (pSUVs)

Integrin was reconstituted in proteoliposomes according to the general procedure developed in Ref. [66]. Typically, a lipid mixture of SOPC and 2% DOPE-PEG was vacuum-dried before being resuspended in MQ water with 1.5% Triton X-100. Then, 10 L of integrin αIIbβ3 at 1.8 mg/mL was added at a protein:lipid ratio of 1:1760. The protein–lipid mixture was incubated at 37 ${}^{\circ }$C for 30 min under agitation, then stored overnight at 4 ${}^{\circ }$C for micelle equilibrium. The next day, Triton X-100 was removed by incubation with 16 mg SM2 Bio-beads under stirring for 3 h. Reconstituted proteoliposomes were separated from the beads by aspiring the supernatant. Before use, the proteoliposomes were extruded 20 times with a 100 nm pore membrane (Whatman, Maidstone, UK) using a mini-extruder apparatus (Avanti Polar Lipids, Alabaster, AL, USA) to obtain a solution of Small Unilamellar Vesicles (SUVs) of controlled size. The SUV monodispersity was measured using dynamic light scattering (DLS) (Zetsizer nanoseries from Malvern, UK). SUV solutions were stored at 4 ${}^{\circ }$C up to 2 weeks. Alternatively, for comparison, the proteoliposomes were sonicated using either a bath sonicator (10 min at 37 ${}^{\circ }$C) or a tip sonicator (Fisher Scientific, Waltham, MA, USA) set to 40% duty cycle with a pulse time of 2 s followed by a rest period of 2 s for a total sonication time of 2 min. In all three cases small unilamellar proteoliposomes were obtained, henceforth referred to as pSUVs. For negative control experiments, small unilamellar vesicles—SUVs—without incorporation of integrins were similarly prepared.

### 2.5. Formation of Giant Unilamellar Vesicles Containing Integrins (pGUVs)

Integrin-containing pGUVs were prepared from integrin-containing pSUVs. As a control, GUVs without integrins were prepared from pure lipid mixture (SOPC + 2% DOPE-PEG) in chloroform or from pure lipid SUVs.

#### 2.5.1. PVA-Assisted Swelling Method

GUVs and pGUVs were prepared using the recently developed PVA-assisted method [55]. PVA solution (5%, *w*/*w*) was prepared by dissolving PVA (polyvinyl alcohol) in MQ water at 90 ${}^{\circ }$C under stirring for 2 h, and stored at RT for up to several weeks. PVA-coated substrates were prepared by spreading 200 L of PVA solution on a coverslip then drying the PVA film for 30 min in an oven at 50 ${}^{\circ }$C. A total of 20 L of lipids in chloroform or 10–15 L of SUV/pSUV solution was spread on the dried PVA film and placed under vacuum overnight to evaporate the solvents. A total of 450 L of sucrose solution at 200 mM was gently deposited on the dried lipid or pSUV layers.

GUV formation was monitored using phase contrast microscopy. When the desired GUV size (10–20 m) was reached, typically in 1 h, GUVs were harvested using a pipette with a cut tip, and transferred into an Eppendorf tube for storage at 4 ${}^{\circ }$C.

#### 2.5.2. Electro-Swelling Method

In addition to the PVA-assisted swelling method, GUVs (or pGUVs) were also prepared using the electro-swelling technique [23,51,60]. Lipids dissolved in chloroform (or pSUVs in aqueous solution) were deposited onto indium-tin-oxide (ITO)-coated electrodes. After evaporating the solvent (same protocol as gel-assisted swelling), a home-made swelling chamber was filled with a 200 mM sucrose solution, and the content was exposed to an AC electric field of 1.7 V at 10 Hz for 3 h. The GUVs (or pGUVs) were harvested and observed as for those prepared by gel-assisted swelling.

### 2.6. Characterisation of SUVs and pSUVs by Cryo-Transmission Electron Microscopy (Cryo-TEM)

A few microlitres of SUV/pSUV solution was deposited on cryo-EM grids covered with a holey carbon film, which was previously made hydrophilic by glow discharge at low air pressure. The grids were then blotted with filter paper and rapidly frozen by plunging them in liquid ethane cooled by liquid nitrogen. After flash-freezing, a vitreous sample layer filled the holes of the carbon film. The frozen grids can be either stored or immediately transferred to a cryo-holder, inserted in the cryo-EM instrument (FEI Tecnai 200 KV), and imaged.

### 2.7. Imaging

GUVs were imaged during the PVA-assisted swelling process using phase contrast microscopy, on a transmission bright-field microscope (Olympus, Japan). Images were taken with a 10× objective, and recorded using a CDD camera (COHU, San Diego, CA, USA). Snapshots were taken at regular intervals of 10 min. After formation, GUVs were introduced into an observation chamber and observed using epifluorescence microscopy, transmission bright-field microscopy, and reflection interference contrast microscopy (RICM) performed on an inverted microscope (AxioObserver, Zeiss, Germany), equipped with an EM-CCD camera (iXon, Andor, North-Ireland). Acquisition was performed using Andor iQ software, and images and time sequences were taken with a 63× 1.25 NA oil antiflex objective (Zeiss), with an exposure time of 100 ms for bright-field and RICM and 200 ms for epifluorescence. Calibration of the images after the acquisition and quantitative analysis of data was done using home-written routines in Image J software or Python programming language.

## 3. Results

**Figure 1:**
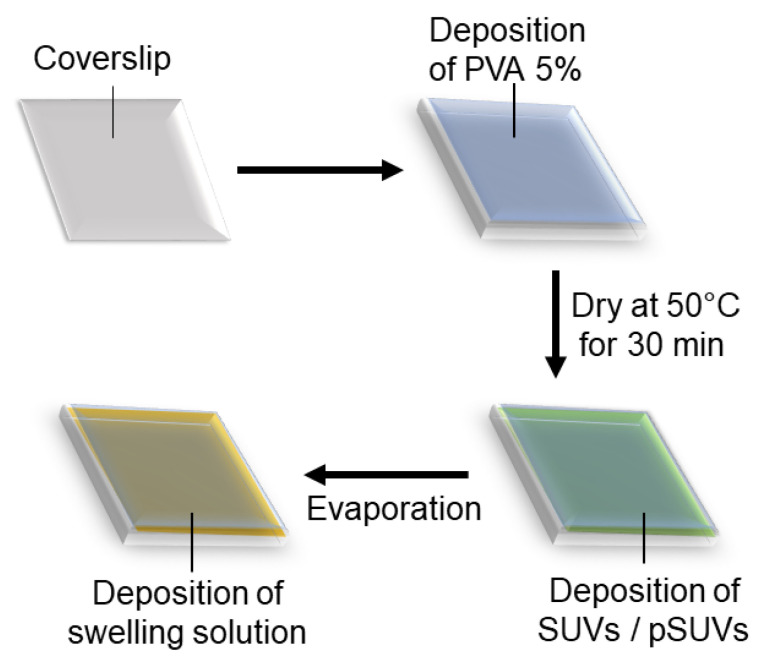
Schematic representation of the protocol for PVA-assisted swelling of GUVs incorporating integrins. A glass coverslip is coated with an aqueous solution of PVA which is dried, then coated with a solution of previously prepared SUVs which is again dried. In the last step, the swelling buffer is deposited. The PVA as well as the dried lipid–protein mixture are hydrated, and GUVs are formed.

GUVs incorporating integrins were successfully prepared using gel-assisted swelling, for which we developed a new protocol. Our protocol combines the classical method for gel-assisted swelling [55] using lipids dissolved in chloroform, with the modified electro-swelling protocol reported for transmembrane proteins such as integrins [60]. Our basic protocol consists of coating a thin layer of PVA (polyvinyl alcohol) with a pre-prepared solution of small unilamellar vesicles containing integrins (pSUVs), drying it and rehydrating it with the desired buffer to swell integrin-containing giant unilamellar vesicles (pGUVs) (Figure 1). Here we shall describe the optimisation of each preparation step and enumerate the conditions which are, according to our experiments, important for maintaining a robust protocol. We characterise the formed pGUVs in terms of their diameter and level of fluorescence, the latter being an indication of the amount of integrin incorporated, and also compare the new method with electro-swelling.

### 3.1. Optimising Gel Preparation

**Figure 2:**
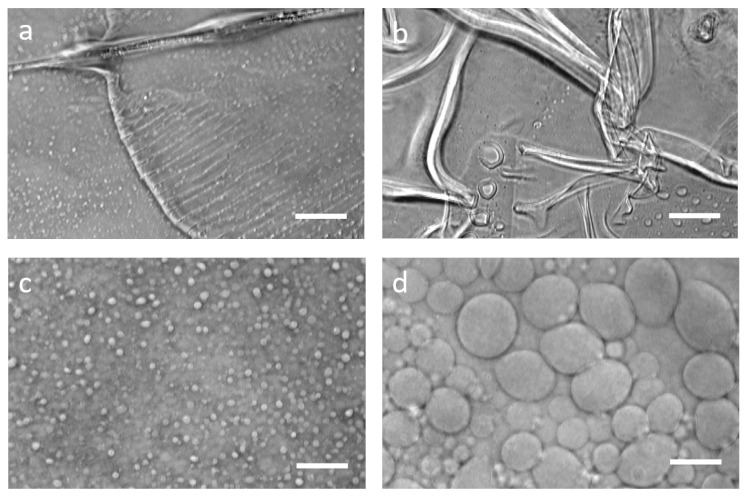
PVA-assisted swelling. Examples of folds (**a**) and cracks (**b**) of the PVA layer, leading to no swelling (if folds) or low-yield swelling (if too many cracks). Images of GUVs during PVA-assisted swelling at t = 2 min (**c**) and t = 15 min (**d**). Scale bars: 10 m.

Our starting point was the original gel-assisted swelling protocol [55], which reported GUV preparation from a variety of lipids dissolved in chloroform. We found that for our conditions, plasma cleaning accompanied by washing with ethanol and ultra-pure water of the glass coverslips was not sufficient, and washing with an alkaline detergent recommended for cleaning quartz cuvettes is a good option (protocol described in the Methods section). The PVA itself also needs to be correctly chosen—a shorter chained 89 kD PVA could not support swelling, and we retained the 145 kD polymer used in the original protocol [55]. The PVA was dissolved in pure water in the original protocol, but several other aqueous solvents, including PBS and sucrose, have been suggested in the literature [55,56,58]. After several trials we found that all of these worked well if the GUVs were swollen from lipid deposited from solution in chloroform. However, when lipids were deposited from an aqueous solution containing SUVs, as is the case here, PVA dissolved in water was optimal. We also encountered the problem of the PVA layer developing folds or cracks, especially if the ambient temperature rose much beyond 25 ${}^{\circ }$C. While a few cracks are not usually a problem, presence of too much cracking prevented successful swelling. It was previously reported that this problem could be overcome by spin-coating the dissolved PVA instead of spreading manually [55]. However, in our hands spin-coating was not beneficial and was dispensed with (Figure 2).

### 3.2. Optimising SUV Preparation

The lipid composition with which we have chosen to work here is SOPC, doped with 2% DOPE-PEG and 2% DOPE-RGD if appropriate. We have also successfully tested a mixture of egg-PC and 2% DOPE-PEG as well as egg-PE and 2% DOPE-PEG using the same protocol and found the protocol to be robust as far as lipid composition was concerned. While optimising the protocol, we found that sometimes it was useful to incorporate up to 4% DOPE-PEG to prevent the finally formed GUVs from sticking to each other and forming clumps. However, in the final optimised protocol, 2% DOPE-PEG was retained, as this is generally sufficient to prevent GUV clumping.

Small liposomes were prepared following standard protocol, and were treated as described in the Methods section. We found that the method of preparation of the SUVs had an impact on the later formation of GUVs. Three methods of preparation of SUVs were tested: tip-sonication, bath-sonication and extrusion. The formed SUVs were characterised by DLS, and it was found that SUVs prepared using either of the two sonication techniques had two populations—one with size around the nominal 100 nm expected and another smaller peak corresponding to large aggregates. In terms of GUV formation, tip sonication and extrusion both gave reasonable yield and with large size of 15 to 20 m, whereas the yield from bath sonication was not satisfactory, and the size was also smaller on average (Table 1). We have retained extrusion as the method of choice, but tip sonication can also be used.

### 3.3. Preparation and Storage of pSUVs

As introduced in the methods section we use the word “proteoliposomes” for the polydisperse small vesicles containing integrins obtained by hydration, which are then used to prepare integrin-containing SUVs that are presumably more monodisperse (pSUVs), which in turn are then used to form GUVs with integrins (pGUVs). As described above, integrin extracted from human platelets was purified using affinity chromatography, labelled with Alexa Fluor 546 at 58% labelling rate, and stored in a buffer containing detergent. We used a protein:lipid concentration ratio of 1:1760 for all experiments reported here. Pure lipid small liposomes, obtained by simple hydration, were functionalised by incubation with the purified integrins solubilised with surfactant and subsequent removal of the surfactant using Bio-beads (see the Methods section for details). We tested a few different surfactant concentrations and found those recommended for electro-swelling of integrin-containing GUVs [60] to be the best. The proteoliposomes were extruded to obtain well-defined pSUVs. For swelling pGUVs, the pSUVs are usually recommended to be partially dried. In our hands, however, whether the evaporation was partial or complete did not impact the pGUV swelling or protein function. The time of evaporation was however important, and at least 8 h of drying under moderate vacuum was necessary. The pressure was found to not be a crucial factor, and a low-pressure vacuum generated by a standard membrane-pump was found to be sufficient.

Temperature throughout the deposition and drying process was found to be an important factor—ambient temperature of more than ≈25 ${}^{\circ }$C was detrimental—shifting the whole procedure to a cold room at 4 ${}^{\circ }$C restored the protocol. Thus, the drying was done overnight, under vacuum, with no need to maintain humidity, but ensuring that a temperature between 4 to 25 ${}^{\circ }$C was maintained. We found that the proteoliposomes can be stored either at −20 ${}^{\circ }$C or at 4 ${}^{\circ }$C with similar shelf life of about 10 days. Beyond the recommended 10 days, the swelling process is less robust and may fail.

### 3.4. Characterisation of SUVs and pSUVs

**Figure 3:**
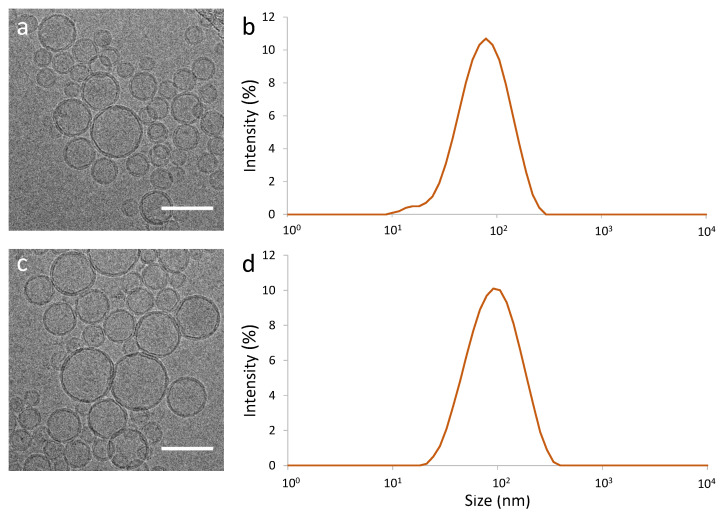
Characterisation of pure lipid SUVs (**a**,**b**) and integrin-containing pSUVs (**c**,**d**) by Cryo-Electron Microscopy (Cryo-TEM) ((**a**,**c**), scale bars: 100 nm) and Dynamic Light Scattering (DLS) (**b**,**d**). At this resolution, the transmembrane protein is not visible in TEM.

We characterised the extruded pSUVs and compared them to pure lipid SUVs using electron microscopy (EM, Figure 3a,b) and dynamic light scattering (DLS, Figure 3c,d). We quantified their size from both EM images and DLS data (Table 2). It was seen that the SUVs were essentially spherical, and their shape and size were not impacted by the presence of the integrins (which are not individually visible in EM at the resolution that we could achieve).

### 3.5. Preparation of GUVs

**Figure 4:**
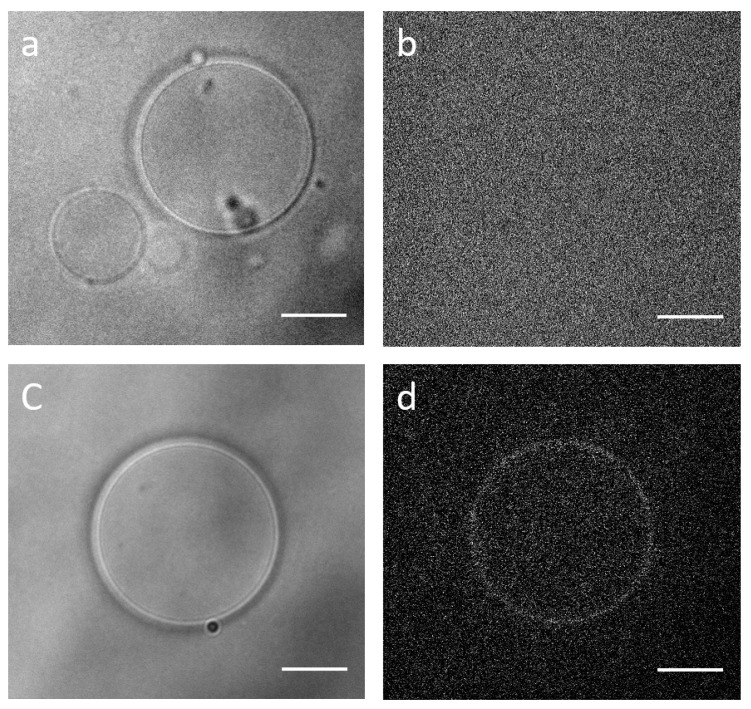
GUVs without integrins (**a**,**b**) and pGUVs containing fluorescent integrins (**c**,**d**) formed by PVA-assisted swelling; bright-field (**a**,**c**) and epifluorescence (**b**,**d**). Scale bars: 10 m.

The final step of swelling was done in sucrose buffer. The formed GUVs were transferred to an observation chamber containing PBS buffer with an osmolarity chosen such that the GUVs could become slightly deflated to facilitate later adhesion assays. In previous sections, we showed that the yield and size of SUVs were not impacted by the presence of the transmembrane protein (Table 2 and Figure 3). Accordingly, it did not affect the swelling of GUVs: whether prepared from SUVs or pSUVs, the average diameter ranged from about 10 to 15 m. Quantification of the diameter of 30 GUVs, swelled the same day and under the same conditions, gave the values 13 ± 8 m for pGUVs with integrins and 13 ± 7 m for GUVs without integrins (Figure 4).

### 3.6. Electro-Swelling vs Gel-Assisted Swelling

**Figure 5:**
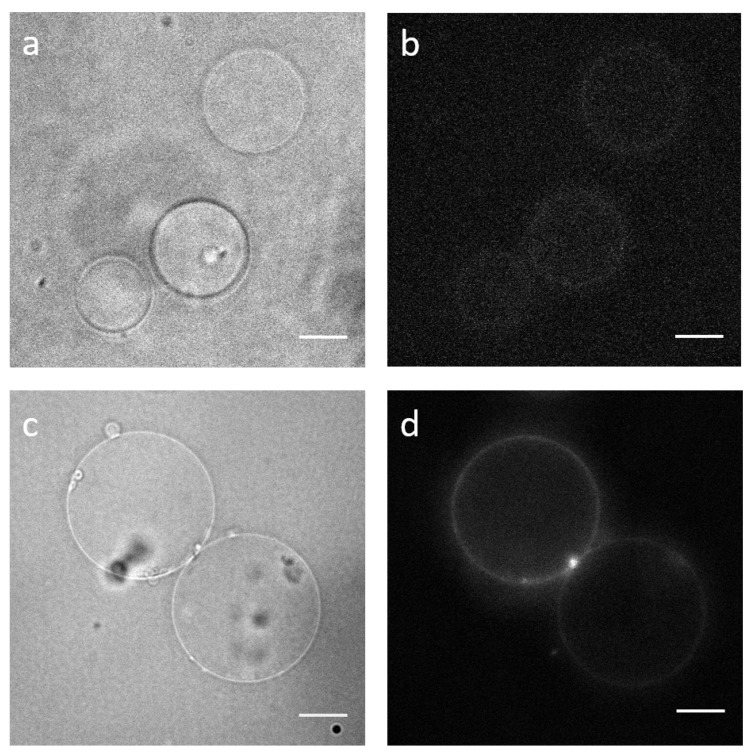
Comparison of pGUVs prepared using electro-swelling (**a**,**b**) and PVA-assisted swelling (**c,d**). Bright-field images of pGUVs (**a**,**c**) and epifluorescence images of incorporated integrins (**b**,**d**). Note that the diameter values reported in the text are population averages, and individual GUVs shown in the figure may be smaller or larger. Scale bars: 10 m.

Electro-swelling is often the benchmark for GUV studies and was previously successfully used to make integrin-containing GUVs. For this reason we compared pGUVs obtained from gel-assisted swelling and from electro-swelling (Figure 5). We found that, for our lipid composition, the electro-swelled pGUVs were, on average, of the same diameter, but fewer integrins were incorporated. Quantification from two different comparable days of experiments, under the same conditions, gave diameters of 19 ± 9 m for electro-swelled pGUVs (average of 36 individuals) and 11 ± 5 for gel-swelled pGUVs (average of 37 individuals). Pooling data from different experiments over an extended period gave 18 ± 11 m for electro-swelled pGUVs and 17 ± 13 m for gel-swelled pGUVs (average of 6 experiments and 140 individuals, Mann Whitney Wilcoxon rank-sum test gives *p* = 0.1, indicating that the populations were not statistically different). Direct comparison of the integrin content is difficult because the electro-swelled pGUVs could not be imaged under the same conditions as the gel-swelled pGUVs (Figure 5). The average intensity of gel-swelled pGUVs was about five times higher than that of electro-swelled pGUVs, even when imaged with a camera gain that was ten times lower (averages done over about 10 individuals in each case). Since the gain factor is not linear, it is unfortunately not possible to estimate the ratio of the number of integrins incorporated.

### 3.7. Adhesion Assay to Test Functionality

**Figure 6:**
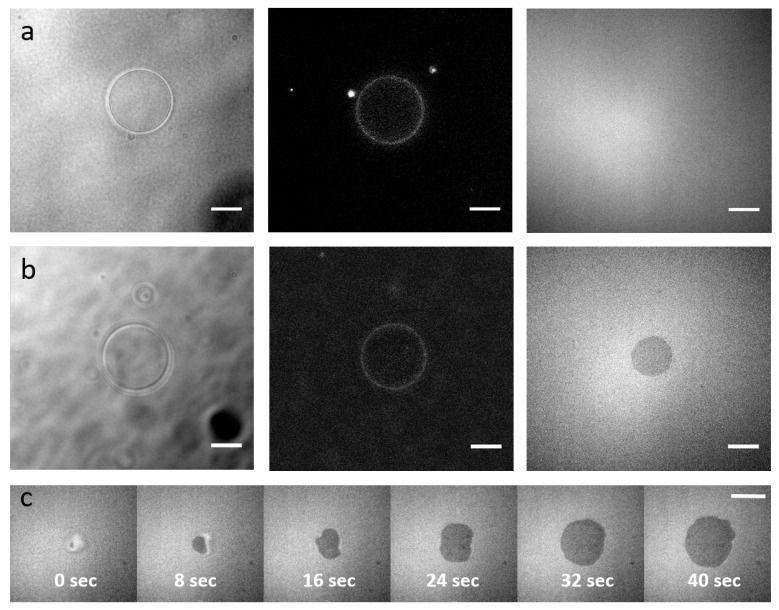
Functionality of the integrins. (**a**,**b**) pGUVs containing integrins are deposited on Supported Lipid Bilayers (SLBs) without RGD ligand of integrins (**a**) or with RGD (**b**). Left: Bright-field channel, GUVs. Middle: Epi-fluorescence channel showing integrin incorporation in the GUV. Right: RICM, dark regions indicate adhesion. (**c**) Timelapse of an integrin containing pGUV adhering to an RGD functionalized SLB (time is indicated). Scale bars: 10 m.

The formed integrin-containing pGUVs were allowed to interact with a supported lipid bilayer (SLB) that was either functionalised with DOPE-RGD or not carrying any ligands for integrins (Figure 6). The presence of integrins in the pGUVs was verified by fluorescence imaging. The presence of DOPE-PEG lipids in the SLB as well as the pGUVs ensured that in absence of ligands, the pGUVs did not adhere to the SLB, thus providing a clear negative control. However, in the presence of DOPE-RGD, the pGUVs adhered to the substrate, forming a dense, roughly circular adhesion disc, as indicated by the dark patch seen in RICM [67,68]. The dynamics of pGUV adhesion could be followed, and it was seen that the entire adhesion process took a few tens of seconds.

## 4. Discussion

We have presented an optimised protocol for gel-assisted swelling of integrin-containing pGUVs. The starting point of the protocol developed here was from previously reported protocols to prepare integrin-containing SUVs [36,60,69,70]. Our choice of protein:lipid ratio was inspired by such previous work. Quantification of integrin and lipid concentration in SUVs [69] showed that for such initial ratios, the final ratio was about 1:7000. Here, we expect a similar incorporation rate. Our choice of lipid was driven by a desire to use a pure synthetic phosphatidylcholine (PC) that remains fluid at room temperature. We chose a lipid with a single unsaturated chain rather than two unsaturated chains because the latter is more unstable to oxidation under storage. We also tested the commonly used egg-PC, which is a mixture of different lipids, as well as the equivalent phosphatidylethanolamine: egg-PE. All the tested lipids could successfully be swelled into integrin-containing GUVs.

We found that the crucial pitfalls to avoid include the control of gel layer stability—too much cracking or peeling-off leads to no GUV formation; control of lipid drying and GUV swelling temperature, which should be in the range of 4 to 25 ${}^{\circ }$C; and SUV clumping, which sometimes prevents GUV swelling. Importantly, we showed that the gel technique is compatible with transmembrane proteins which need to be dissolved in an aqueous solution rather than chloroform. We see that at the high integrin-to-lipid ratio presented here, the integrins are better incorporated into gel-swelled GUVs as compared to electro-swelled ones. The integrin molecules are homogeneously distributed on the gel-swelled GUV membrane, though the amount of incorporated protein may vary by a factor of two from one experiment to another under the same conditions. We showed that the incorporated integrins are functional in terms of specific adhesion. This protocol now opens the possibility of obtaining GUVs in high-salt, physiologically relevant buffer, and encapsulation of other actin binding proteins and signalling molecules.

## Figures and Tables

**Table 1:** Impact of different SUV preparation techniques on their size and their ability to make GUVs. SUV size was characterised by dynamic light scattering (column DLS). The width of the peak is reported as error, and the reported PDI is in parenthesis. * indicates presence of a much smaller second peak corresponding to a larger object size, indicating presence of aggregates. GUVs formed using each of these category of SUVs was characterised in terms of size using bright-field microscopy (column BF). Diameter reports averages from experiments using the same batch of SUVs as DLS (between 10 and 20 GUVs were measured for each case). Average diameter reports averages from 3 different days (at least 10 GUVs each). The standard deviation is reported as error.

Technique	DLS (SUVs)	BF (GUVs)	BF (GUVs)
	Size ± Width in nm	Diameter	Average Diameter
	(PDI)	± s.d (m)	± s.d (m)
Tip Sonication	107 ± 73 (0.352) *	17 ± 7	16 ± 7
Extrusion	85 ± 44 (0.215)	14 ± 3	14 ± 4
Bath Sonication	128 ± 86 (0.334) *	7 ± 3	10 ± 4

**Table 2:** Comparison of measured size of SUVs with integrins (pSUVs) or without (pure lipid SUVs), using cryo-TEM or DLS. The two types of SUVs have similar sizes, and the values obtained from the two techniques agree within the error bars. For TEM images, >100 individuals were measured. Error is standard deviation for cryo-TEM data and peak-width for DLS data.

	Cryo-TEM	DLS
	Diameter ± s.d. (nm)	Size ± Width in nm (PDI)
With integrin (pSUVs)	79 ± 25	107 ± 48 (0.217)
Without integrin (SUVs)	60 ± 18	85 ± 44 (0.215)

## Data Availability

Not applicable.
